# Genetic mechanism for the loss of PRAME in B cell lymphomas. Reply.

**DOI:** 10.1172/JCI161979

**Published:** 2022-07-15

**Authors:** Katsuyoshi Takata, Christian Steidl

**Affiliations:** 1 Centre for Lymphoid Cancer, British Columbia Cancer, Vancouver, British Columbia, Canada.; 2 Division of Molecular and Cellular Pathology, Niigata University Graduate School of Medical and Dental Sciences, Niigata, Japan.; 3 Department of Pathology and Laboratory Medicine, University of British Columbia, Vancouver, British Columbia, Canada.

**Keywords:** Hematology, Immunology, Cancer, Lymphomas, Molecular pathology

## The authors reply:

We thank Dr. Mraz for insightful comments that provide additional context to the findings in our study, in particular bringing to the forefront studies in other B cell malignancies including chronic lymphocytic leukemia (CLL) (1). As pointed out in the Letter by Dr. Mraz and discussed in our manuscript, PRAME deletions were significantly associated with Ig-λ rearrangements, and this finding is consistent with a mechanism in which PRAME deletions can occur in the context of rearrangement of variable (V) gene loci on chromosome 22 in a subset of patients (2). For this reason, we studied frequencies and treatment outcomes for PRAME deletions in the context of Ig-λ expression, as shown in Figure 1 (2). In particular, we demonstrate that PRAME deletions are independently associated with outcomes in multivariable analysis, making it unlikely that PRAME deletions are a pure surrogate for the prognostic effects of Ig-λ usage. As described by Mraz and Pospisilova, the association of PRAME deletion and Ig-λ rearrangement and expression is not absolute, and PRAME deletion is likely dependent on the exact V segment usage (3). Consistently, we did not observe PRAME deletions in the majority of patients expressing Ig-λ, and, interestingly, a minority of patients with PRAME deletions expressed Ig-κ. Indeed, it remains an open question whether heterozygous or homozygous PRAME deletions can occur during the process of B cell lymphomagenesis, and potentially at the time point of the germinal center (GC) reaction in GC-derived diffuse large B cell lymphomas (DLBCLs). Regardless of the deletion-generating mechanism, our study brings into focus PRAME loss–associated cell-autonomous and tumor microenvironment phenotypes that are under selective pressure in DLBCL. Our discovery that both genomic PRAME deletion and EZH2 mutations converge on reduced PRAME expression and downstream phenotypes underscores the pathogenic relevance of the PRAME gene in B cell lymphoma.

## Version 1 07/15/2022

Electronic publication

## Version 2 07/18/2022

Text error corrected: lambda symbol changed to kappa
